# Comparison of the TLR4/NFκB and NLRP3 signalling pathways in major organs of the mouse after intravenous injection of lipopolysaccharide

**DOI:** 10.1080/13880209.2019.1653326

**Published:** 2019-08-25

**Authors:** Qin Gong, Luling He, Mulan Wang, Shasha Zuo, Hongwei Gao, Yulin Feng, Lijun Du, Yingying Luo, Jun Li

**Affiliations:** a School of Pharmaceutical Sciences, Jiangxi University of Traditional Chinese Medicine, Nanchang, China;; b State Key Laboratory of Innovative Drugs and Efficient Energy-saving Pharmaceutical Equipment, Jiangxi University of Traditional Chinese Medicine, Nanchang, China;; c College of Pharmacy, Guangxi University of Chinese Medicine, Nanning, China;; d School of Life Sciences, Tsinghua University, Beijing, China

**Keywords:** LPS, varied inflammatory reaction, intestine, kidney, liver, lung

## Abstract

**Context:** Lipopolysaccharide (LPS) is often used to induce immunoinflammatory reactions. TLR4/NFκB and NLRP3 signalling are major factors for inflammation. Dexamethasone (DXM) has an anti-immunoinflammatory effect.

**Objective:** To investigate the inflammatory reaction in pathological changes of organs and the expression of inflammatory signalling during LPS infection.

**Materials and methods:** ICR mice were divided into control group (*n* = 9), LPS group (*n* = 15) and LPS + DXM group (*n* = 14). LPS (10 mg/kg) was injected intravenously in LPS group and LPS + DXM group, normal saline was injected to the control group; DXM (0.5 mg/kg) was given by intragastric administration. 12 h after LPS, the blood was collected and the organs were isolated for biochemical analysis, protein expression, and morphological examination.

**Results:** The results showed that BUN, Cre, ALT, AST in the LPS group increased distinctly by 81.42, 67.84, 40.53 and 36.05%, respectively, and CK, ALP, TP and ALB decreased by 71.37, 60.6, 12.57 and 19.73%, respectively, compared with the control group. In the morphologic observation, local necrosis in the liver, arterial vasodilation in the heart and kidney, alveolar secretions and pulmonary interstitial in the lungs, and mucosal shedding in the small and large intestines, the expression of TLR4-NFκB signalling were up-regulated distinctly whereas NLRP3 signalling was less broadly affected. DXM can decrease BUN and Cre, downregulate the expression of TLR4-NFκB signalling, but has no effect on the organ damage based on morphology.

**Conclusion:** Acute injuries induced by LPS are extensive. The inflammatory damage in small and large intestines, liver and kidney was more severe than other organs. TLR4-NFκB signalling was the major response to LPS stress.

## Introduction

Lipopolysaccharides (LPS) is a mucopolysaccharide produced by Gram-negative bacteria (Rosner et al. 1979; Cote and Taylor 2017). LPS is also a major factor that causes cell damage during the bacterial invasion of the body. LPS can effectively activate the body’s innate immune system and cause inflammatory reactions (Yoshioka et al. 2009). Therefore, LPS is often used as an alternative to cell infection models in experimental studies (Tobias et al. 1988; Pedron et al. 1994) and LPS simulates damage to the body that is caused by bacteria.

For *in vivo* studies, LPS is often administered by injection, such as intraperitoneal and intravenous injections. In addition, other methods are employed depending on the organ, including intranasal or aerosol inhalation for lung treatment and intracerebroventricular (ICV) administration for brain treatment. Intravenous administration, however, is a diverse method for establishing injuries in different organs, such as in an experimental model for observing heart damage (Piechota-Polanczyk et al. 2012) or an experiment to observe lung injury (Ali et al. 2018), as well in experimental models for observing damage to the kidneys, spleen, brain, liver, and intestines (Szot et al. 2017; Li H et al. 2018; Pervin et al. 2018; Stoyanoff et al. 2018; Wu et al. 2019). This motivates the question of why different organ injury models exist for the same intravenous drug delivery route: are other organs damaged in the process of establishing the organ injury model, and is there a problem with model-specificity? This is a problem that needs to be addressed.

Studies have shown that LPS as a ligand can activate toll-like receptor 4 (TLR4) on the cell membrane, initiate the expression of the downstream inflammatory response signalling pathway, and finally, through the upregulation of the inflammatory or necrosis factors IL-1β (interleukin-1β), IL-6 (interleukin-6) and TNFα (tumor necrosis factor α), produce an inflammatory response (Chow et al. 1999). NLRP3 (NOD-, LRR- and pyrin domain-containing 3) is an inflammasome, which is another cellular inflammatory response pathway (Davis et al. 2011). Its high expression often triggers a chronic inflammatory response process (Mangan et al. 2018). Since the TLR4 pathway and the NLRP3 pathway constitute the LPS-triggered inflammatory reaction process, in this work, the protein expression of modulators in these two signalling pathways is used to measure the inflammatory responses of major organs after LPS infection in order to explore the extent of organ damage within this acute LPS infection model and provide accurate experimental data for new drug research.

## Materials and methods

### Animals

Male ICR mice weighing 21–23 g were purchased from Hunan SJA Laboratory Animal Co., Ltd. [SCXK (Xiang) 2016-0002]. This experiment was completed in the Laboratory of Barrier Environment of the Jiangxi Bencao-Tiangong Technology Co., Ltd. [SYXK(Gan)2018-0002]. The mice (10 mice in a large cage) were observed in the animal room for 3 days before the experiment. The temperature of the room was controlled at 25 °C and the relevant humidity was controlled at 50–60%. The experimental research program was approved by the Experimental Animal Ethics Committee of Jiangxi University of Traditional Chinese Medicine (Approval number: JXLLSC-2018-33). The experimental procedure strictly followed the guidelines of the Experimental Animal Welfare and Ethics of China.

### Reagents and instruments

LPS (Lipopolysaccharides from *Escherichia coli* O555: B5, L2880-100MG) was purchased from Sigma (USA) (Batch number: 017M4112V). Dexamethasone (DXM) was purchased from Anhui Golden Sun Biochemical Pharmaceutical Co., Ltd. (Anhui, China) (batch number: 15032521). 7100 Automatic biochemical analyzer (Hitachi, Japan), Bio-Rad electrophoresis unit (USA), and Bio-Rad ChemiDocXRS + Gel Imaging System (USA) were used in this study. LEICA RM2235 paraffin slicer (Germany) was used to generate sections for microscopy. LEICA DM2500 Optical Microscope (Germany) was used to measure neutrophil invasion. Urea nitrogen (BUN) (R1 TG836, R2 TG837), total protein (TP) (TH619), albumin (ALB) (TF126), aspartate aminotransferase (AST) (R1 AR792, R2 TG862), alanine aminotransferase (ALT) (R1 AR794, R2 TH622), lactate dehydrogenase (LDH) (R1 AR796, R2AP318) and alkaline phosphatase (ALP) (R1 TF168, R2 AR800) were all purchased from Japan Pure Pharmaceutical Industry Co., Ltd. in Shanghai, China. Creatinine (Cre) (710241 H) and creatine kinase (CK) (708021 G) were purchased from Beijing Leadman Biochemical Co., Ltd. (Beijing, China).

### Mouse model

The ICR mice were randomly divided into three groups: the control group (9 mice), the LPS group (15 mice) and the LPS plus DXM group (LPS + DXM) (14 mice). The LPS modeling dosage (10 mg/kg, i.v.) was chosen based on the results from Li T et al. (2018) and was modulated by our prelimited experimental results. Six hours after the injection of LPS, DXM was given in the LPS + DXM groups, the dose of dexamethasone was 0.5 mg/kg (administered by intragastric administration), which is the mouse equivalent of the clinical dose, the control group and the LPS group were given equal volumes of normal saline. Blood was taken from the infraorbital veins of mice 6 h after administration and serum was separated at 3500 rpm for 10 min, the biochemical indices of AST, ALT, BUN, Cre, TP, ALB, LDH, CK and ALP were examined using an automatic biochemical analyzer. The mice were sacrificed using anesthesia and the organs were isolated, half of the heart, liver, spleen, lung, kidney, brain, small intestine and large intestine of each mouse was stored at −80 °C for measuring protein expression, and the rest of the organs were fixed in 4% formaldehyde solution for histological examination.

### Protein expression

Protein expression was analyzed by western blotting as previously described (Chai et al. 2014; Yuan et al. 2015). For western blot analysis, primary antibodies against NFκB (rabbit polyclonal antibody, ab16502), TNFα (rabbit polyclonal antibody, ab6671), NLRP3 (rabbit polyclonal antibody, ab210491), IL-1β (rabbit polyclonal antibody, ab9722), IL-6 (rabbit polyclonal antibody, ab208113) and caspase1 (rabbit polyclonal antibody, ab1872) were purchased from Abcam (UK). TLR4 (mouse monoclonal antibody, sc-293072) and ASC (mouse monoclonal antibody, sc-271054) were purchased from Santa Cruz (USA). IL-18 (rabbit polyclonal antibody, TA324190) was purchased from ORIGENE (USA). The goat anti-mouse IgG-HRP (ZB2305) and goat anti-rabbit (ZB2301) IgG-HRP secondary antibodies were purchased from ZSGB-Bio, Beijing, China. The targeted proteins were visualized with the Super Signal West Femto Chemiluminescent Substrate (Thermo Scientific Pierce) and the intensity of the visualized bands were analyzed using Quantity One software (Bio-Rad). β-actin (mouse monoclonal antibody, TA-09, Zhongshan Jinqiao Biotech company, Beijing, China) was used as an internal control. Data are expressed as a ratio to β-actin.

### Morphological analysis

After 6 days of formalin fixation, the tissues were thoroughly rinsed with water then embedded in paraffin wax and stained with hematoxylin and eosin (HandE) using a standard protocol (Wang et al. 2015) The tissues were observed under a light microscope. The histopathological diagnoses were performed by two different research scientists independently. All images were recorded at 200× amplification.

### Data analysis

The data obtained are expressed as the mean ± SD. The chart data was processed using GraphPad Prism 5.0 software (USA). Each dataset was analyzed using a one-way ANOVA for the *F*-test and then a *t*-test between groups. A *p*-value of less than 0.05 was considered to be a statistically significant difference.

## Results

### General manifestation of mice after intravenous injection of LPS

At 12 h post-LPS injection, the mice in the LPS group showed tiredness and less movement than the normal group of mice, and some mice had secretions from the nose and no thin stools or bleeding. Others did not show a specific response. DXM provided some improvement, but an overall effect was not obvious.

### Changes in the serum biochemical index after LPS injection

**Figure 1.**
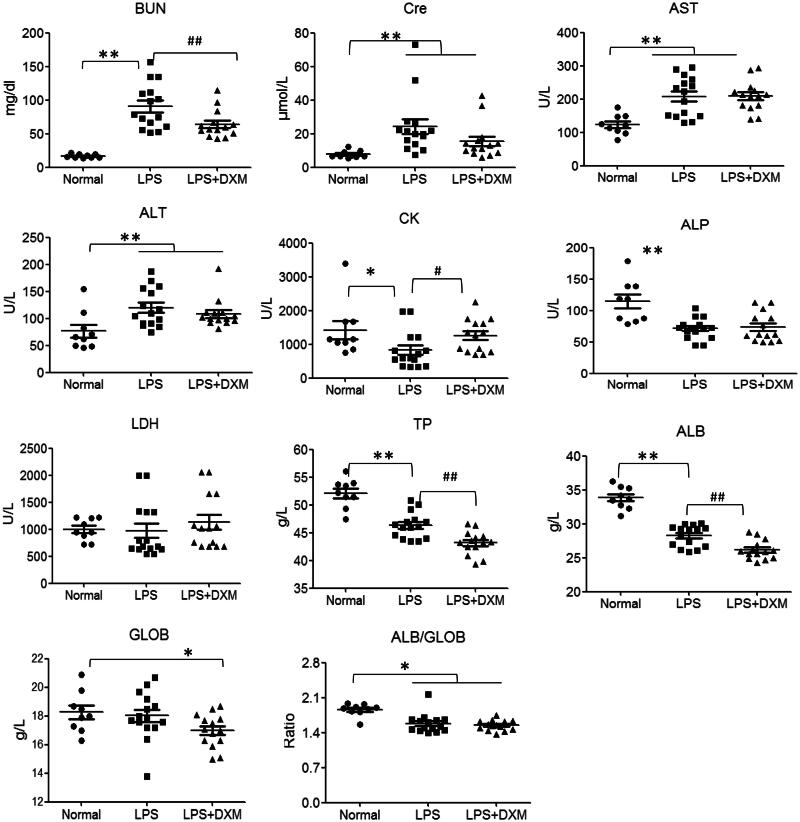
Parameters of biochemistry of serum samples of mice after intravenous administration. Dexamethasone (DXM) was used as 0.5 mg/kg. Data are presented as the mean ± SD (9 mice in the normal group, 15 mice in the LPS group and 14 mice in the LPS + DXM group). *p < 0.05 or **p < 0.01 *vs.* normal group. #*p* < 0.05 or ##*p* < 0.01 *vs.* LPS group.

After the LPS injection, the main mouse serum biochemical indicators were significantly changed. Among them, the levels of BUN and Cre, which correspond to renal function, were significantly higher in the LPS group than the normal control group. The levels of AST and ALT, which indicate liver function, were also significantly higher in the LPS group than in the normal control group, suggesting LPS damage to the liver and the kidney. In addition, CK and ALP, which reflect the stress response and general body damage, were also significantly decreased, indicating that there was extensive LPS damage to the body. Notably, the serum TP, ALB levels of LPS-infected mice were significantly decreased, and the A/G ratio (ALB/GLOB) was significantly lower in the LPS group than in the normal group, reflecting liver damage. As a classical and effective anti-inflammatory immune drug, DXM has obvious inhibitory effects on renal damage, restoring the stress response indicators CK. However, the serum ALB, TP were decreased significantly, but had no effect on GLOB and A/G and, an effect on liver function was not observed (Figure 1).

### Expression of TLR4/NFκB signalling

**Figure 2.**
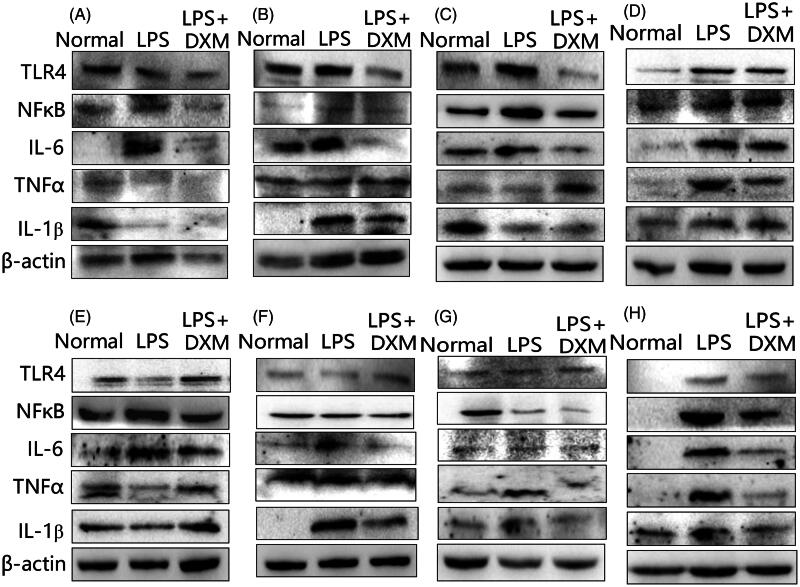
Images of protein expression of the TLR4/NFκB signalling pathway in the major organs of mice after intravenous administration of LPS. Western blot assays were employed. (A) Heart; (B) liver; (C) spleen; (D) lung; (E) kidney; (F) brain; (G) small intestine; (H) large intestine. DXM: Dexamethasone.

**Figure 3.**
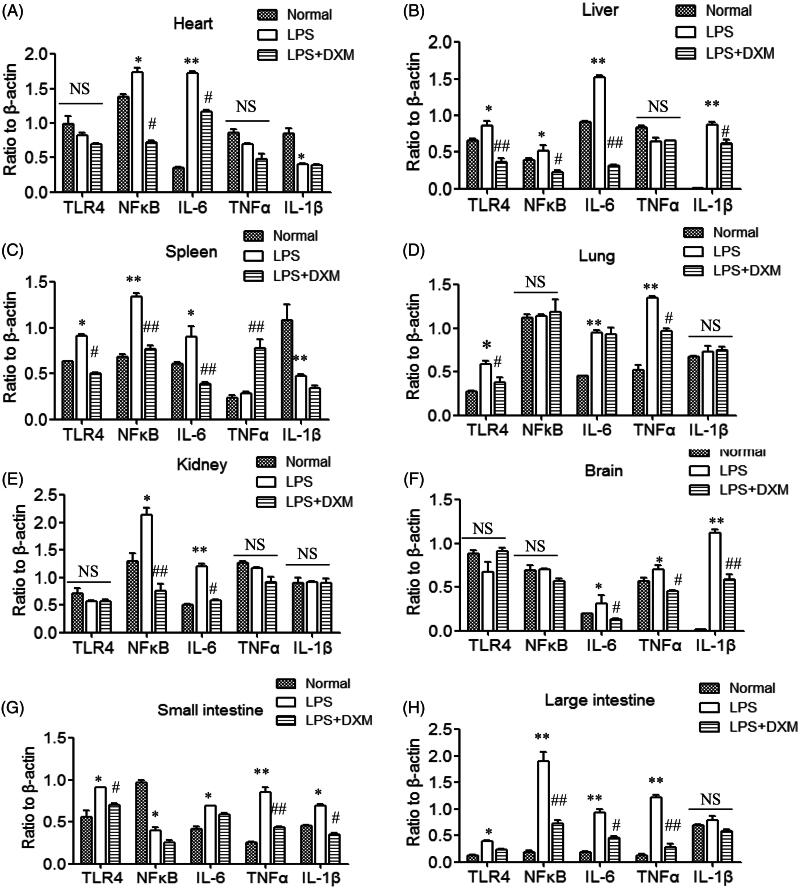
Protein expression of the TLR4/NFκB signalling pathway in the major organs of mice after intravenous administration of LPS. DXM was used as 0.5 mg/kg. Data are presented as the mean ± SD (9 mice in the normal group, 15 mice in the LPS group and 14 mice in the LPS + DXM group). *p < 0.05 or **p < 0.01 *vs.* normal group. #*p* < 0.05 or ##*p* < 0.01 *vs.* LPS group. NS: no significance.

Figures 2 and 3 show that the expression of inflammatory factors in various organs was upregulated after intravenous injection of LPS. The most obvious response was that the expression levels of TLR4-NFκB in the large intestines and its downstream signalling proteins IL-6, TNFα and IL-1β were significantly upregulated, suggesting activation of this pathway. In the small intestines, TLR4 and IL-6, TNFα and IL-1β were also expressed at significantly higher levels. The expression of NFκB/IL-6 was upregulated in the heart, and the expression levels of TLR4-NFκB/IL-6 and IL-1β were upregulated in the liver. In the spleen, the expression of TLR4-NFκB/IL-6 was upregulated, and the expression of TLR4/IL-6 and TNFα was upregulated in the lung. In the kidney, there was no significant change in TLR4 expression, but the NFκB/IL-6 expression was upregulated. There was no significant change in TLR4 or NFκB upstream of brain signalling, but the expression of downstream inflammatory factors TNFα, IL-6 and IL-1β was upregulated. This suggests a difference in the inflammatory responses of the major organs after LPS intravenous administration. The DXM group displayed inhibition of this upregulation to some extent.

### Expression levels of NLRP3 signalling mediators

**Figure 4.**
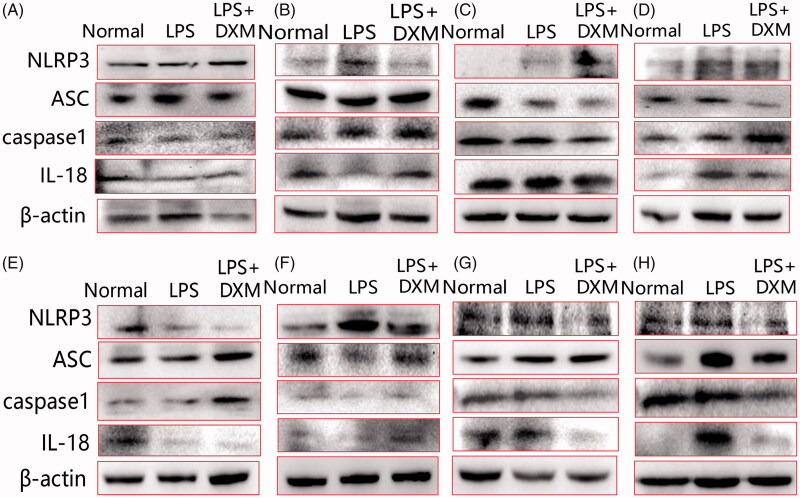
Images of protein expression of the NLRP3 signalling pathway in the major organs of mice after intravenous administration of LPS. Western blot assays were employed. (A) Heart; (B) liver; (C) spleen; (D) lung; (E) kidney; (F) brain; (G) small intestine; (H) large intestine. DXM: Dexamethasone.

**Figure 5.**
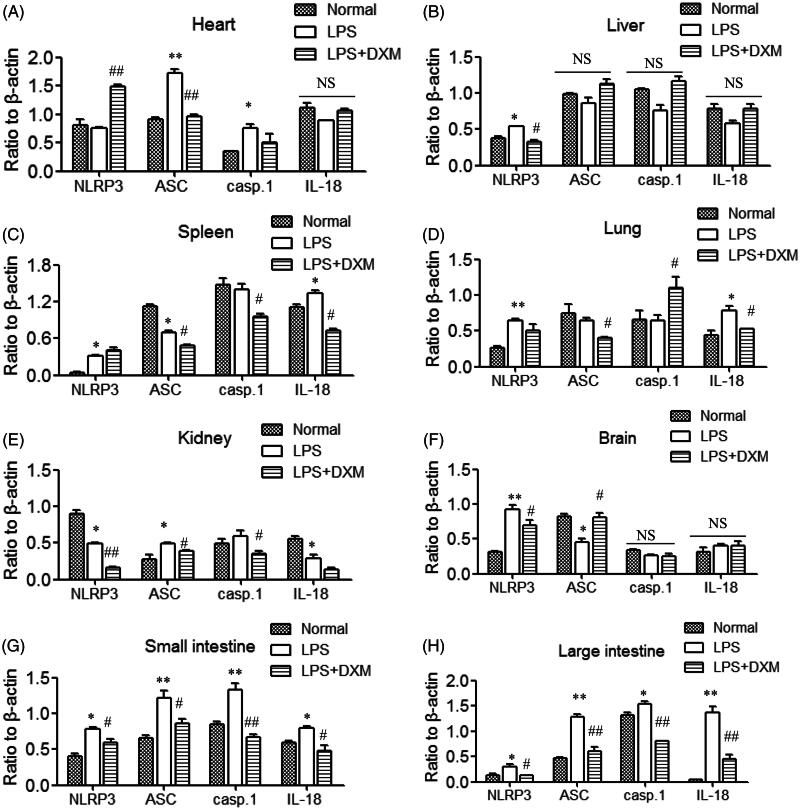
NLRP3 protein expression in the major organs of mice after intravenous administration of LPS. DXM was used as 0.5 mg/kg. casp.1: caspase1. Data are presented as the mean ± SD of mice (9 mice in the normal group, 15 mice in the LPS group and 14 mice in the LPS + DXM group). *p < 0.05 or **p < 0.01 *vs.* normal group. #*p* < 0.05 or ##*p* < 0.01 *vs.* LPS group. NS: no significance.

The NLRP3 pathway consists of NLRP3, ASC, caspase1, IL-18 and IL-1β. After intravenous injection of LPS, the mediators of the NLRP3 pathway were upregulated in different mouse organs. The protein expression levels in the small intestine and large intestine were the most distinct, and the expression of the mediators of the NLRP3-ASC-caspase1/IL-18 pathway was upregulated. In the heart, ASC-caspase1 expression was obviously increased. Only NLRP3 expression was elevated in the liver, but no significant response was observed in the downstream signals. The expression level of NLRP3 was obviously increased in the spleen, and IL-18 was slightly upregulated. In the lung, NLRP3/IL-18 expression was upregulated, and the expression of ASC in the kidney was elevated. The expression of NLRP3 was significantly elevated in the brain, but no downstream signalling response was observed. Therefore, except for the large intestine and small intestine, the other organs did not respond significantly to the expression of the NLRP3 signalling pathway in LPS-induced acute injury. DXM had an inhibitory effect on this increase in expression (Figures 4 and 5).

### Morphology of the major mouse organs

**Figure 6.**
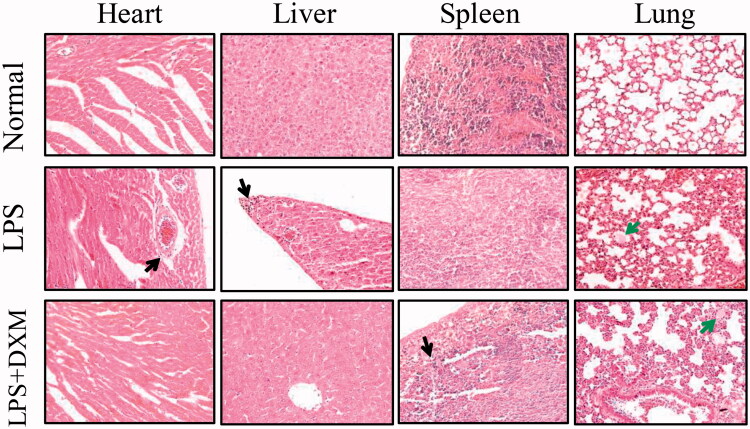
Morphology of the heart, liver, spleen and lung after intravenous injection of LPS (200× amplification). DXM: dexamethasone. Black arrow indicates the neutrophil infiltration and green arrow indicates alveolar secretion.

**Figure 7.**
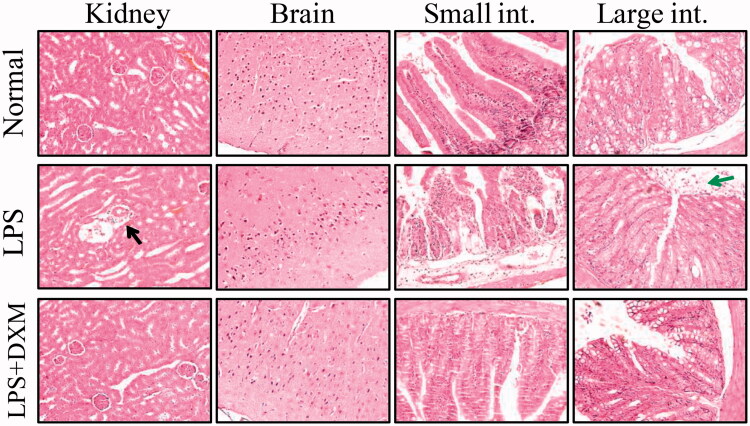
Morphology of the kidney, brain, small intestine (small int.) and large intestine (large int.) after intravenous injection of LPS (200× amplification). DXM: dexamethasone. Black arrow indicates neutrophil infiltration and green arrow indicates relapsed mucosa.

In the histological analysis, there were different inflammatory responses within the major mouse organs following the intravenous injection of LPS. This reaction mainly manifested as neutrophil infiltration and degeneration and necrosis in local areas. There also appeared to be local necrosis in the liver. Arterial vasodilation was found in the heart and kidney. Alveolar endocrine secretions and pulmonary interstitial thickening were observed in the lungs. In the small intestine and large intestine, mucosal shedding was found. There were no distinct injuries to either the heart or brain. DXM did not significantly inhibit local inflammatory responses (Figures 6 and 7).

## Discussion

Our study reveals that, in the acute injury induced by LPS injection, the TLR4-NFκB pathway is stimulated in response to the inflammatory reaction. In general, the acute injury induced by LPS is mainly driven by TLR4 because LPS is the TLR4 ligand (Kawasaki et al. 2000). However, an increase in the expression of NLRP3 pathway mediators has also been reported to result from the acute injury induced by LPS (Li H et al. 2018; Zhao et al. 2018). The NLRP3 pathway is part of the cellular inflammasome. It can be activated by various factors such as intracellular short-chain fatty acids and oxygen free radicals (Yuan et al. 2018). This inflammasome signals an inflammatory response and ultimately releases IL-1β and IL-18 (Coll et al. 2016). Studies have shown that NLRP3 is mainly found in chronic inflammatory processes and other noninfectious pathological processes such as Alzheimer's disease (Wu et al. 2017; Anders et al. 2018; Hoseini et al. 2018; Qi et al. 2018; Yang et al. 2018). TLR4 can activate NLRP3 through intermediate factors (Gurung et al. 2015). Recent studies have reported that TLR4 receptor activation can ultimately promote NLRP3 expression and the assembly of inflammatory bodies through CMPK2 effects on mitochondrial DNA (Murphy 2018; Zhong et al. 2018). These data suggest that there is a close relationship between TLR4 and NLRP3. However, our results indicate that NLRP3 is not significantly stimulated, suggesting that the large-scale response to the acute injury induced by LPS is related to TLR4, not NLRP3. This may be a result of the shorter, 12 h period of our experiment. LPS activates TLR4 via downstream signal expression or directly activates NFκB expression, which, in turn, causes immune-inflammatory responses (Zhang et al. 1999).

Serum biochemical analysis of acute LPS infection over 12 h revealed that the liver and kidney function-related indicators BUN, Cre, ALT and AST were increased, suggesting that the liver and kidney had been damaged. However, no obvious lesions were observed in the histology of the liver and kidney, but a few samples showed neutrophil infiltration or cell necrosis in local areas. NFκB/IL-6 protein expression in the liver and kidney showed significant upregulation, in agreement with the literature (Shi et al. 2017). It can be concluded that this injury is mainly caused by an acute inflammatory reaction. Notably, serum ALB and TP decreased significantly after the LPS injection compared with the levels in normal mice, indicating that LPS inhibits hepatocyte synthesis of ALB. In the early literature, there are several reports of LPS inhibition of ALB synthesis by hepatocytes, but these studies were conducted with cell-based *in vitro* experiments (Wang et al. 2005a). The findings suggest that LPS could inhibit the expression of ALB mRNA via the P38 MAPK signalling pathway (Wang et al. 2005b) or that LPS is correlated to the activation of NFκB (Wang et al. 2004). Our work is the first to demonstrate a decrease in serum ALB and TP after LPS injection *in vivo*. DXM has been reported to promote the synthesis of ALB by hepatocytes (Nawa et al. 1986); however, DXM in our study did not have an obvious antagonistic effect on LPS inhibition of ALB and TP. This indicates that DXM, in such a small dose, cannot antagonize the inhibition of protein by LPS infection. There might also be specific LPS inhibition of ALB synthesis. This subject warrants further study.

The effect of LPS on the brain was weak in our study, which may be related to a blocking effect by the BBB (blood-brain barrier). In addition to intravenous injections in the literature (Nordgreen et al. 2018), intra-cerebro ventricular (ICV) administration is often used when LPS affects brain function and nerve cells (Nakano et al. 2015). However, it has also been reported that an inflammatory reaction is observed after intraperitoneal administration (Liu et al. 2015; Lykhmus et al. 2016). Further experimentation is needed.

In this work, the intestinal inflammatory response caused by intravenous LPS was found to be more pronounced, in agreement with what has been reported by Zuo et al. (2010). However, there are few reports on intestinal injury *in vivo*, most of which is observed in cultures of small intestinal epithelial cells and small intestinal tissues *in vitro* (Hori et al. 2001), and there are few reports on the large intestine. Our results revealed that LPS can also cause immune-inflammatory reactions in the colon, which should be a reference for the screening of drugs for the prevention and treatment of colon diseases.

Pulmonary infections are usually treated by intranasal or aerosol inhalation or by direct instillation of the trachea so that treatments are directly exposed to lung tissue (Fonceca et al. 2018; Jiang et al. 2018; Lee et al. 2018; Zhang W et al. 2018; Zhang X et al. 2018) because acute lung injury is mostly a process of no more than 12 h. However, intraperitoneal injection of LPS was often used in subacute experiments, which were observed for 3 weeks after LPS exposure (Dong and Yuan 2018). Our experimental results show that the lung also produces acute immune inflammatory responses after intravenous injection, as well as an interstitial inflammatory reaction, mild inflammatory cell infiltration, increased alveolar endocrine secretion and other pathophysiological changes that are weaker than local exposure in general. Therefore, our work suggests that if one only seeks to observe lung damage, it is better to use local exposure.

Our experiments also indicate that myocardial damage is mild after LPS injection. There are reports of heart rhythm or ventricular dysfunction and the expression of related factors caused by LPS both *in vivo* and *in vitro* (Nemoto et al. 2002; Panaro et al. 2010; An et al. 2012; Unuma et al. 2013). However, it can be seen from our results that in the acute process, myocardial damage is not apparent during the 12 h observation time, and the expression of related inflammatory factors is not particularly notable. Except for the increased expression of NFκB, IL-6, and ASC, there was no significant upregulation of inflammatory factors. Therefore, the LPS acute injury model in the short-term application of this intravenous injection should be used with caution when screening cardioprotective drugs.

After exposure to LPS, the protein expression of TLR4-NFκB/IL-6 in the spleen of mice was significantly increased, but exposure had no significant effect on the signalling of the NLRP3 pathway, suggesting that this acute inflammatory response is dominated by the TLR4 signalling pathway. The effects of LPS on the spleen inflammatory response have been reported in mice, rats, and guinea pigs, and the route of administration is mostly intravenous injection (Feleder et al. 2003; Gorąca et al. 2013). Most of these studies observed the spleen immune-inflammatory response and other related immune factors (Xiao et al. 2015), as well as the activation of the spleen dendritic immune cells (Xu et al. 2017). Since the spleen is closely related to the body's immune cells, this LPS-induced spleen inflammatory response can be used for the screening of related immunoinflammatory inhibitor drugs.

From the study of LPS infection effects on the heart, liver, spleen, lung, kidney, brain, small intestine and large intestine, it can be seen that this damage is systemic. Therefore, this should be carefully considered when choosing a model for observing damage to a particular organ. Strictly speaking, intravenous injection of LPS is a systemic infection process that is similar to bacteremia/toxemia in the body. The endpoint result is damage to all organs of the body until functional failure. Therefore, in addition to the observation of specific organs, other important organs should be investigated as much as possible in the screening of new drugs. In addition, it should be noted that the expression of mediators of the TLR4 pathway in the large intestine and small intestine showed a consistent increase 12 h after the intravenous injection of LPS, which provides an important experimental basis for the study of new intestinal infection drugs.

Taken together, these data reveal the changes in serum biochemistry of the whole body and the protein expression of related inflammatory factors within major organs after intravenous injection of LPS. The acute injury caused by LPS infection over 12 h was mainly an inflammatory reaction and clearly the response of the TLR4-NFκB pathway. Among the inner organs, the expression of inflammatory factors in the large intestine and small intestine was upregulated distinctly. The acute injury caused by LPS is systemic because of the intravenous administration. Therefore, it is also necessary to detect the changes within other organs when focusing on specific organs so that the medicinal effects of candidate drugs can be more comprehensively evaluated.
